# Modelling the Influence of Composition on the Properties of Lightweight Plaster Mortar and Multicriteria Optimisation

**DOI:** 10.3390/ma16072846

**Published:** 2023-04-03

**Authors:** Khrystyna Moskalova, Tatiana Lyashenko, Aleksej Aniskin, Matija Orešković

**Affiliations:** 1 Department of Processes and Apparatuses in the Technology of Building Materials, Odessa State Academy of Civil Engineering and Architecture, 4 Didrihsona St., 65029 Odesa, Ukraine; 2 Department of Information Technologies and Applied Mathematics, Odessa State Academy of Civil Engineering and Architecture, 4 Didrihsona St., 65029 Odesa, Ukraine; 3 Department of Civil Engineering, University North, 104. Brigade 3, 42 000 Varaždin, Croatia

**Keywords:** plaster mix, viscosity, thixotropy, strength, design of experiment, experimental–statistical model, multicriterial optimisation

## Abstract

The influence of the components of plaster mortars on their properties is considered in a lot of studies at a qualitative level without searching for optimal compositions of these multicomponent composite materials. The purpose of this study was to obtain the experimental–statistical models based on the results of the designed experiment, allowing the influence of light fillers and polymer admixtures on the properties of the mortars to be evaluated and analysed; the compositions complying with specified requirements and compromised optimally by a number of properties should be found. The quantities of fine limestone and perlite as well as of the hydroxyethyl methyl cellulose and dispersible polymer were varied in the experiment. The effective viscosity and thixotropy of the mix, compression, tensile, adhesive strength, frost resistance, and density of hardened mortars were determined for 18 compositions according to the experiment design. The obtained models have allowed the individual and synergetic effects of mix components to be evaluated. The fine perlite has turned out to have the greatest positive effect on the properties. This porous filler increases the strength while decreasing the density of the mortars. It is shown how the composition complying with specified requirements—and the best based on several properties—has been found.

## 1. Introduction

The goal of material science is to make high grade materials that perfectly match their intended purpose. Therefore, they are generally referred to as High Performance Materials [1,2,3], with composites being their special class. Both high-strength concrete for bridge structures and low-strength quick-hardening mortar for short-term fixing of rocks can be used as building composite materials. In order to create building composites which would serve their purposes to the fullest extent, multi-component components are used. What can be called the “nested” multicomponent is characteristic of binder systems, complex admixtures, poly-fractional fillers, hybrid fibres, etc. [4,5,6]. The plaster mortars considered below are the composites of multicomponent disperse systems.

Nowadays, the studies devoted to the improvement of the properties of these materials have shifted towards the use of fine dry additives [7,8,9,10]. Admixtures are supplied as powders, the typically used low dosages being 0.3–1.5% of the weight of the cement. The researchers have established [11,12,13,14] that the introduction of additives improves workability and lowers the water demand of the mix, confers air entrainment, or adds waterproofing qualities. These changes have the potential to decrease cracking through improvements in the paste–aggregate bond and to increase the strength of hardened mortars. The type of additive is a critical aspect governing its effect on cementitious mixes. 

Because of the increasing necessity for reliable heat insulating protection of building structures, the functions of the heat-insulating layer are performed by lightweight plasters. Such mixes have an average density of ≤1300 kg/m^3^ [15] and decreased thermal conductivity, creating a homogeneous and uniform thermal resistance over the entire surface of the wall, excluding the so-called “thermal bridges”, which are formed in the masonry joints and on the joints of fastening heat-insulating materials. Currently, lightweight fillers (expanded perlite, limestone powder, etc.) are widely used for mortars and made to reduce the heat transfer coefficient [16,17]. However, due to the porous structure, perlite and limestone can lead to a decrease in physico-mechanical characteristics, so the choice of the optimal amount of lightweight aggregates is an important aspect of this study.

This work is a part of an extended study of [18], and its purpose is to better understand the behavior of lightweight mortars containing lightweight aggregates and admixtures in order to optimize mortars properties. The mortar mixes should have certain rheological properties such as fluidity, stability, and viscosity, which affect and define the workability, pumpability, self-leveling, and compacting. Thixotropy is particularly significant for plaster mortars [18]. When using the mixes on a vertical surface, it is essential to impede their slipping and provide its original structure. On the other hand, the mixing process and type of mixer can influence the mortar’s properties, in particular the rheological parameters [19,20,21,22,23,24]. It is important to control particle homogenisation and dispersion in mixes, but there are no commonly used methods to carry out such studies. Studying the mixing process would be more effective with the help of various rheological characteristics [25,26,27,28,29].

The introduction of fine limestone powder into the cementitious mixtures increases the yield stress and plastic viscosity of cement paste [30]. Likewise, the rheological behavior of the mortars changes with the type and content of fine limestone, as well as by including or excluding the admixture [31]. Furthermore, Costa et al. [31] demonstrated that the tensile bond strength of the mortars diminished when the limestone content was raised. Still, bond strength can be enhanced by the presence of dispersed fine particles and suitable rheological behavior.

As reported in the study [13], a material with beneficial technical and technological properties that are relevant for mortars administered by machine can be obtained due to the rheology of plastering mortars with hydrated lime and cellulose ether. The authors of [32] have shown that the presence of cellulose ethers in hydraulic lime-based mortars may decrease the density of mortars in a plastic state and improve their frost resistance due to higher open porosity. As has been noted in [33], the increase in the contents of cellulose ether and of lime results in a reduction in compressive strength and adhesion. Hence, it is important to know that wrong quantity of cellulose ether may lead to the loss of important physico-technological properties. This is necessary to find the optimal dosage of cellulose in the presence of hydrated lime. 

It has been also noted [34] that an increase in the ethylene-vinyl acetate (EVA) copolymer in mortar decreased the dry density, while the density of the mortar with an EVA content of 5% was similar to the control (0%). This occurred because of the air-entraining effect of the EVA, which led to higher porosity. In addition, a small amount of EVA can enhance the flexural and compressive mortar strength. Nevertheless, this study showed the effect of EVA in individual presence, while other additives are usually also present in addition to EVA in mortars. Therefore, in our study, we investigate both the individual effect of EVA and its joint effects with other commonly used additives. Such a comprehensive assessment of the mortars gives the right view in relation to the tested one.

The publications mentioned above give useful information (important for the “picture” as a whole) about the influence of components on this or that property. However, quantity dependencies are relatively seldom. Sometimes, so-called optimal compositions present just the result of the choice between the two options.

In order to achieve desirable properties of composite materials at separate phases of their life (mix, forming structures, hardened composite, degrading material to be utilised), the relations of the properties with multi-component composition and process (CP) parameters (with operational conditions) have to be quantitatively examined.

The CP parameters, with various set levels, stand for the vector of CP factors, ***x*** = (*x*_1_, …, *x*_k_). The criteria of material behaviour (*Y*, generally named properties) are technological and structural features, functional properties, other quality criteria, and any other responses (including resources and costs) to variations of controllable inputs *x*. Additionally, of course, to determine the composition and modes, which would allow us to form the desired structures, which would result in the specified or upgraded levels of the properties, mathematical models to describe the relations between factors *x* and criteria *Y* are necessary.

In some studies of building materials where the design of experiment [35] was used, some regression models were built (not always). However, the obtained data were not always used effectively, in full degree.

In particular, the 9-point Taguchi design was used in the study presented in a very informative paper [36]. The values of rheological characteristics were determined for nine grouts in which all three composition factors varied on three levels. However, “composition–property” functions were not obtained (or at least not shown). The paper [13] describes the study based on the three-factor, three-level Box–Bhenken design of the experiment. Neither the three-factor models with interaction effects nor the optimal contents of the three components are given. In the article [37], it is surprising that there are no interaction effects among the 11 in the models, for which the 27-point experimental design was implemented.

When optimization problems are stated, the task generally involves just one optimality criterion. It may be one of the properties or CP-factor (concretely, minimized should be the quantity of an expensive component or the process parameter that prolongs accomplishing certified material). The task can be formulated with a specific “main” criterion as an objective function, with all other *Y* as constraints. As the only optimality criterion, the integral criterion with weighting multipliers can be used as well, such as with the desirability function [38,39]. The latter is generally used in engineering design when various, often conflicting criteria play a role. The weights are assigned for every criterion. The use of the desirability function is the most popular method [40,41,42]. 

Still, the necessity to assign weights to selected criteria is the primary drawback of the method. The “stiffness” of the decision and the lack of options to flexibly alter the priorities in the process of the search are important disadvantages as well. As observed well in [43], the potential favourable solutions could “get lost due to enforced weighting of the multiple objective functions”. The alternative is to look for the compromise (Pareto optimal solution [43,44,45]), which could be executed without any preference data.

The methodologies of experimental–statistical modelling and of the property fields in composition and process coordinates, developed by V.A. Voznesensky with his co-authors [46,47,48,49], present the informative and effective means (by experimental expenses and the quantity of information mined from experimental data) for obtaining and analysing the “composition–property” dependencies and for solving a great variety of optimisation problems.

It is these methods and tools that have been used in the study presented below.

The aim of the study has been to get a sufficiently flowing mix with high thixotropy and a lightweight hardened mortar of sufficient strength and with good adhesion.

This paper can be considered a sequel of the paper [18] devoted to the rheological characteristics of the plaster mixes.

The aim of the paper is, firstly, to show the levels of a number of physical and mechanical characteristics of hardened lightweight mortars, determined in the designed experiment, and to present the models of the dependencies of the properties on the composition obtained for these data as well. It seems natural to show the influence of the components on the properties, estimated (“measured”) and analysed due to the models, whilst noting the synergistic effects of the components also.

Secondly, it seems important to show the way the multicriteria problem of optimizing the mortar composition can be solved with ES models and the found optimal compromise composition.

A more general goal is to demonstrate some abilities of the methodologies of experimental–statistical modelling and of the composition–process fields of material properties in computational materials science, and thus to promote the application of these helpful methods.

## 2. Materials and Methods

### 2.1. Characteristics of Materials

In this study, we tested mortars based on the following materials. 

As a binder, the additive-free cement M500 mark was used (PC I-500-N D0), European quality certificate EN-197-1 [50], CEM I 42.5N; the specific surface is 300 m^2^/kg and fineness is 11.3%; the ground lime content of CaO + MgO—73% weight, water demand 70%, and bulk density—0.5 kg/dm^3^. 

As an aggregate, the quartz sand from the Volnogorsk Mining and Metallurgical Combine without dust and impurities was used, European quality certificate EN DIN 12904 [51]; the study used sand sifted through a sieve of 0.63. The chemical composition of the quartz sand is given in Table 1.

As fillers, the ground limestone with specific surface S_s.d_. = 400 m^2^/kg was used and sifted through a sieve of 0.63 mm. The chemical and mineralogical composition of the limestone is presented in Table 2. Another filler used in the study was the expanded perlite sand from the Beregovsky quarry of the Transcarpathian region, quality certificate DSTU B V.2.7-157:2011 [52]. Fraction 0.16–1.05, porosity of granules 34.6%, average density (including pores) 1.56 g/cm^3^. The chemical composition of the perlite sand is given in Table 2.

As chemical additives, the additives produced by the Shin-Etsu and Wacker companies were used. Tylose MH60010 [53] water-retaining additive—methyl hydroxyethyl cellulose. Vinnapas RE5034 N [54], adhesion-improving additive. Hostapur OSB [55], air-entraining additive. Vinnapas 8031H [54], water repellent. The physical and chemical properties of the additives are summarized in Table 3.

### 2.2. Design of Experiment

**Figure 1:**
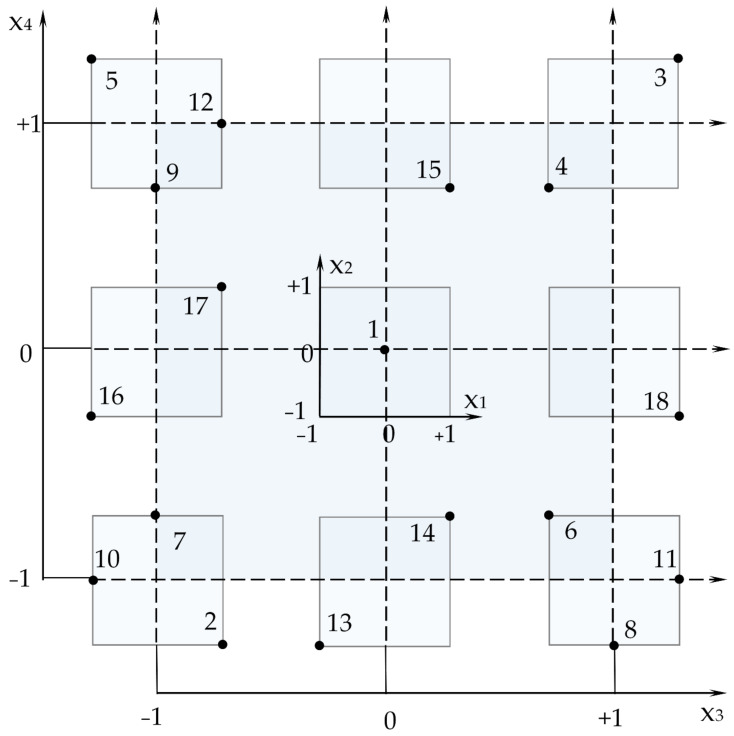
Points of the experiment design.

Four composition factors, the dosages of four components (weight parts, w.p., in 1000 w.p. of dry mix) were varied in the experiment: limestone (marked *X*_1_), perlite sand (*X*_2_), cellulose methyl ether Tylose MH60010 (*X*_3_), and dispersible polymer Vinnapas 5034N (*X*_4_). The contents of other components were invariable. The values of material properties Y were established for 18 compositions in line with 18-points of the 4-factor, 3-level experiment design. The design has been synthesised using the D-optimality criterion [35] with the necessary central point of the experiment. It has already been used in some studies, including the one presented in [18]. The points of the design in coordinates of composition factors normalized to dimensionless −1 ≤ *x*_i_ ≤ +1 are shown in Figure 1, and the natural levels of the factors (dosages of components, dimensional *X*_i_, *X*_i.min_ ≤ *X*_i_ ≤ *X*_i.max_) corresponding to the normalized values are given in Table 4.

This design of the second-order models enables a quantitative description of the data collected; the individual and joint effects of composition factors on properties *Y* using second-order polynomial experimental-statistical (ES) models [46] were of kind (1), where coefficients *b* have a specific physical sense.

(1) $$Y\left(x\right)=b_{0}+\underset{i=0}{\sum }b_{i}x_{i}+\underset{i=1}{\sum }b_{ii}x_{i}^{2}+\underset{i<j}{\sum }b_{ij}x_{i}x_{j}\;\;,\;\;\;$$

where *b* are the parameters (coefficients) to be estimated,

***x***—vector of normalized factors,

*x*_i_ = (*X*_i_ − *X*_0i_)/Δ*X*_i_, *X*_0i_ = (*X*_i.min_ + *X*_i.max_)/2, Δ*X*_i_ = (*X*_i.max_ − *X*_i.min_)/2, and

*X*_i_ = *x*_i_ · Δ*X*_i_ + *X*_0i._

The main steps of building ES models, the specific features and advantages of experimental-statistical modelling, were briefly presented in the previous article [18] and in more detail in the literature cited there. Such models obtained for the properties of the mortars under study are shown below, as well as how useful they can be.

### 2.3. Methods of Testing

The mixing of all mortars was carried out as follows.

- First of all, there was an accurately measured amount of all dry ingredients using the laboratory scale; after this, all dry components were thoroughly mixed with a spatula to ensure a good distribution of all components.
- The water was added into the dry mix and mixed for 60 s with a hand mixer at low speed.

**Figure 2:**
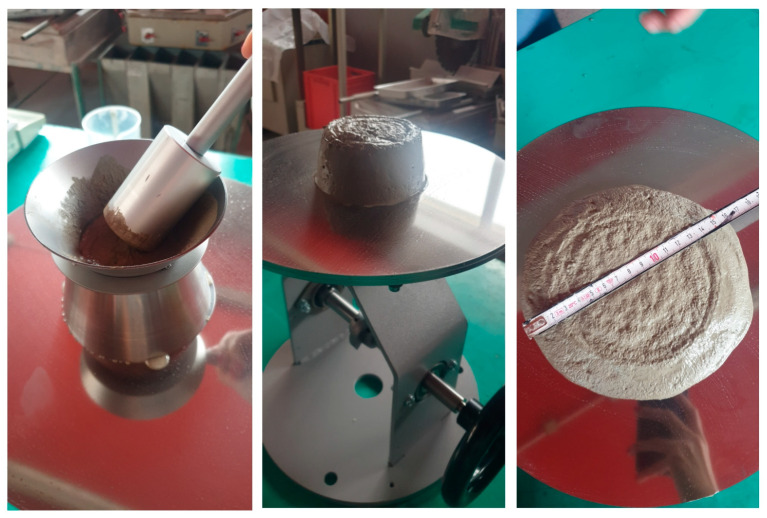
Fresh mixture testing to assess the consistency using the 16–17.0 cm diameter of the spread.

An amount of water was controlled in order to obtain mortars with the same consistency using the diameter of the spread (Figure 2) from the Hagerman cone (16–17.0 cm) according to DIN 18555 [56]. 

**Figure 3:**
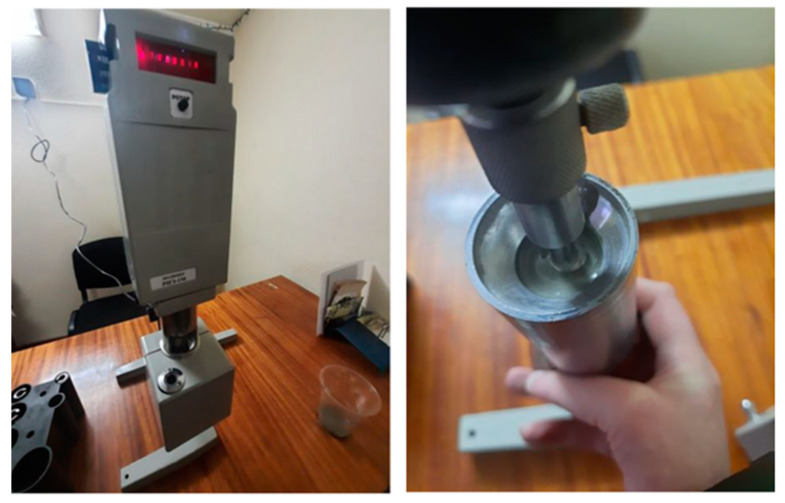
The mixed specimens during the test in the rotational rheometer RPE-1M.

Immediately after mixing the fresh mortar, the samples were put into the rheometer and the measurements were performed. A rotational rheometer RPE-1M, as seen in Figure 3 (made by “Himpribor-1”, Russia), with a coaxial cylinders measuring system according to DIN 53.019 [57] was utilized for the purpose of detecting the rheological properties [58]. This is a rheometer with a driven inner cylinder (the rotor) and measurement of the viscosity-related torque. The external cylinder was kept at the thermostatic chamber and remains at rest; the temperature of the mortar during the measurements was 19 ± 1 °C. The outer cylinder has an inner diameter that equals 24 mm. The inner cylinder has an outer diameter that equals 17.5 mm. The test was run in eight steps with a speed range from 0.045 s^−1^ to 5.705 s^−1^ and repeated, with a repetition of each speed three times. The average value of the data collected during the experiment was taken as the measurement of the effective viscosity η (Pa·s). The duration of the measurement for each sample was 8 h. As a measure of thixotropy, the index *A*_th_ (W_t_/m), equal to the area between upper and lower viscosity curves [18], was determined.

The Ostwald–de-Waele Equation (2) was used to express the viscosity curves of mortars in certain ranges of the shear rate:

(2) $$\eta =K\cdot {\left(\gamma \prime \right)}^{m},$$

The coefficient *K* is equal to the effective viscosity η_1_, Pa∙s, at shear rate γ′ = 1 s^−1^, and the exponent *m* < 0 represents the rate of fluid structure destruction during shear deformation; the higher |*m*|, the less stable the fluid structure during flow [58].

The parameters *K* and *m* in (2), which outline a specific disperse system with a liquid phase (fixed composition), are designated as the so-called “constants” of the physical model. Still, the values of these parameters of the rheological behaviour of the technological mix are determined by its composition, as indicated in the logarithmic form (3) of Equation (2):

(3) lnη (x)=lnK (x)+m(x)· ln γ’,   

Part of the mixture was used to make the specimens 40 mm × 40 mm × 160 mm for determining the properties of hardened mortars. The specimens were continuously cured in a laboratory with a temperature of approximately 23 °C and relative humidity of 60 ± 5%; after removing from molds, the specimens were kept in the same conditions until reaching an age of 28 days. The following mechanical properties were determined: density, tensile and compression strength, and frost resistance. Another part of the mix was applied on the concrete surface to determine adhesive strength.

The density was determined on samples of beams with a size of 40 × 40 × 160 mm, which, after 28 days of hardening at an air temperature of 20 ± 2 °C and a relative humidity of 65 ± 5%, were dried to a constant weight in an oven. Then, according to the obtained results of the geometric dimensions and masses of the samples, the density of the samples was determined.

**Figure 4:**
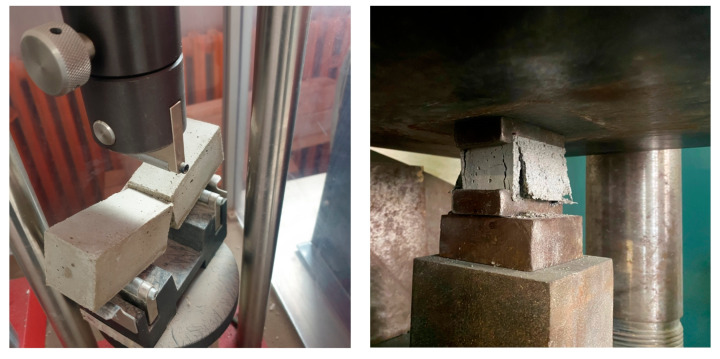
Determination of tensile and compression strength of hardened plastering mortars.

Tensile and compression properties of hardened mortars were tested following the procedures of EN 1015-11:2020 [59]. Three specimens for every composition, according to the experiment design, were tested (Figure 4). After the rupture of the specimens, the mechanical resistance was estimated. The average data were accepted as the measurement of the tensile strength (*f*_t_, MPa). The remaining specimens after testing of tensile strength were used for the determination of compressive strength (*f*_c_, MPa), using a hydraulic press according to the same standard (Figure 4).

**Figure 5:**
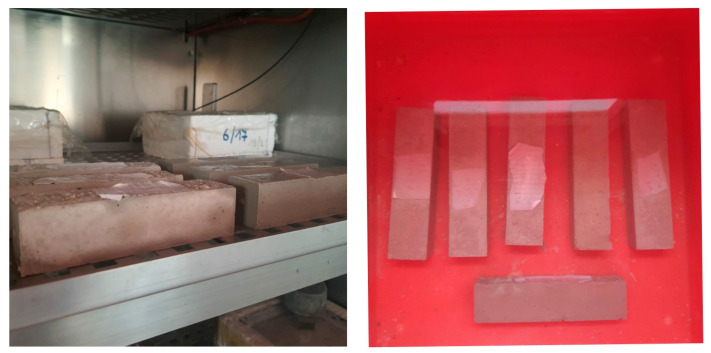
Determination of frost resistance. Specimens in a freeze chamber and in water for thawing.

The CEN/TS 12390-9:2006 [60] was used in the determination of frost resistance. However, some modifications were made, considering the types of investigated materials. Frost resistance was determined by repeatedly alternating freezing and thawing of the specimens, which had prisms of 40 × 40 × 160 mm (Figure 5). The specimens were water-saturated at 18 ± 2 °C and placed in a freeze test chamber, not less than 10 mm aside from each other and at least 20 mm from the walls of the chamber. The temperature was lowered to −8 °C for 8 h. After 8 h of freezing, samples were removed from the freeze chamber and placed in water at 18 ± 2 °C for thawing. Such a cycle type was chosen in order to stimulate the damage in the samples but not to simulate realistic frost effects on mortars [61,62]. Samples were weighed and observed to determine weight loss and defections such as spalling and cracking before each new freezing cycle. The frost resistance was evaluated by weight loss after conducting a certain number of freezing and thawing cycles (*FT*, c.). The permissible value of the mass loss of the samples after alternate freezing and thawing is not more than 5%.

**Figure 6:**
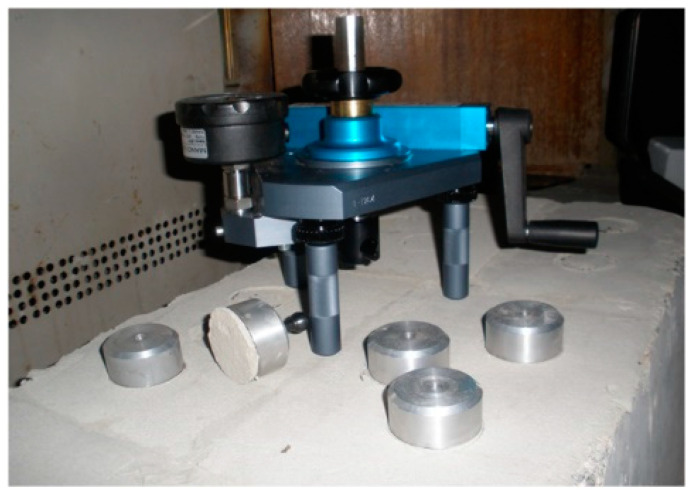
The specimens under adhesion strength tests using DYNA Z16 pull-off tester.

The adhesive strength at 28 days was measured following the procedures of EN 1015-12 [63] and using DYNA Z16 pull-off tester, designed to determine the adhesion strength of stucco mixtures with an accuracy of 0.001 MPa. The mixes were applied on a concrete surface, and after 26 days of hardening the epoxy adhesive was applied on the samples to glue steel pull-heads for testing the adhesion strength (*f*_u_, MPa). The adhesion strength was determined after the adhesive had dried and the samples had reached 28 days (Figure 6).

## 3. Results

### 3.1. Effects of Varied Components on the Properties of the Mortars

The values of hardened mortars properties under consideration, determined for 18 compositions, are given in Table 5 together with two rheological characteristics (given earlier in [18]).

Nonlinear ES models of kind (4), describing “composition–property” dependences, have been built on these experimental data, in particular the one for compression strength (with statistically insignificant effects eliminated at experiment error 0.37%) written in the structured form of (4).
(4)
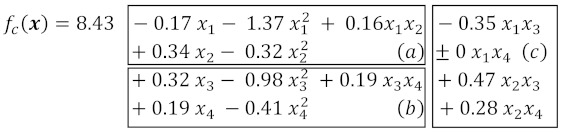


The free term *b*_0_ illustrates the level of the strength at medium values of all *x*, equal to zero, at the center of the experiment. Block (*a*) assesses the effects of the fillers (fine limestone and perlite) at median dosages of polymer additives. The effects of the latter with median contents of the fillers are given in block (*b*). Block (*c*) determines the synergetic (antagonistic) effects of the fillers and additives.

Analogical ES models for all properties under consideration are presented by coefficients *b* in Table 6, including two rheological characteristics considered earlier in [18].

As can be noted in Table 5 only 5 compositions from 18 are critically lost the weight before 50 cycles of freezing and thawing. A characteristic feature of these five compositions is that they contain the lowest or average amount of Tylose and Vinnapas. Obviously, *FT* is sensitive to the presence of admixtures methyllcelulose (*x*_3_) and vinylacetate (*x*_4_) and less sensitive to porous fillers. Coefficients of ES models (Table 6), *b*_3_ = 13.33, and *b*_4_ = 10.65 confirm the positive influence of admixtures on frost resistance. 

The models describe the fields *Y*(*x*) of the material properties in composition coordinates [48,49]. Shown in Table 7 are some numerical characteristics, generalizing indices (*G*) of the fields of the properties in the compositions’ domain, estimated using the models. These are the main results from numerous possible *G:* minimal (*Y*_min_) and maximal (*Y*_max_) levels of the field, their coordinates (*x*{*Y*_min_} and *x*{*Y*_max_}), and absolute and relative increases (Δ_Y_ and δ_Y_).

The models “measure” and allow us to visualise and analyse the individual and joint influence of four components on the properties of the mortars.

The comparison of the generalising indices of the properties (Table 7) shows a significant influence of composition on the adhesive strength; *f*_u_ changes almost six times, while the *f*_c_ and *f*_t_ change two times. With regard to frost resistance, the *FT*_max_/*FT*_min_ ratios are 5.9 and the greatest *FT* = 143 c., which correspond to composition with a high amount of Tylose and Vinnapas.

**Figure 7:**
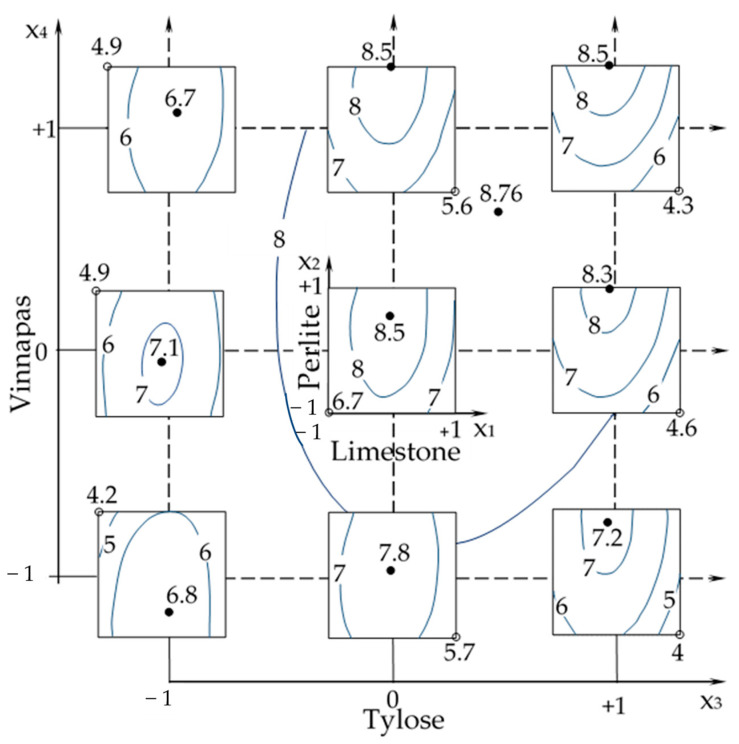
The isolines of *f*_c_ (MPa) in the coordinates of the limestone and perlite contents (9 small squares) changing with dosages of Tylose and Vinnapas; the isoline of the maximum due to the fillers (on small squares) is shown on the “carrying” square.

The diagram “squares on square” in Figure 7 displays the joint influence of four composition factors on the compression strength. As is indicated in Table 7 and can be seen in Figure 7, the highest levels of *f*_c_ come near 8.8 MPa at the highest content of perlite (*x*_2_) and Tylose (*x*_3_) and at a rather high dosage of Vinnapas (*x*_4_). The high values of the individual effects of these components and their synergistic effects can be also noted in Equation (4) and in Table 6 (coefficients *b*_2_, *b*_3_, *b*_4_, *b*_23_, *b*_24_).

The effect of perlite on strength can be explained by the formation of close particle packing and the creation of strong monolithic films. Furthermore, the pozzolanic reaction between perlite and calcium hydroxide can take place, which could lead to the increasing of durability [64,65]. The perlite sand increases mixed thixotropy as well; this can be seen from Table 6, where *b*_2_ = 84.3.

**Figure 8:**
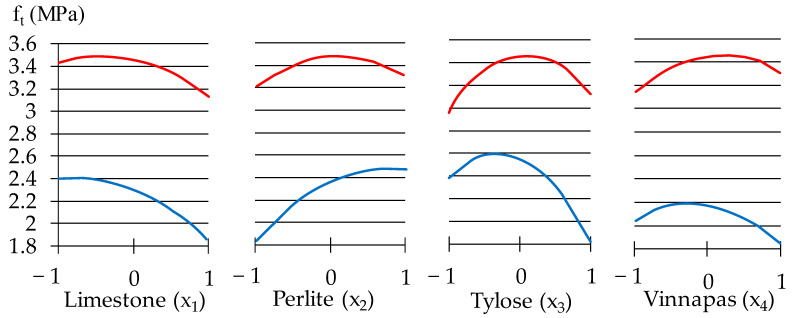
The individual effects of component content on tensile strength in zones of its minimum and maximum.

**Figure 9:**
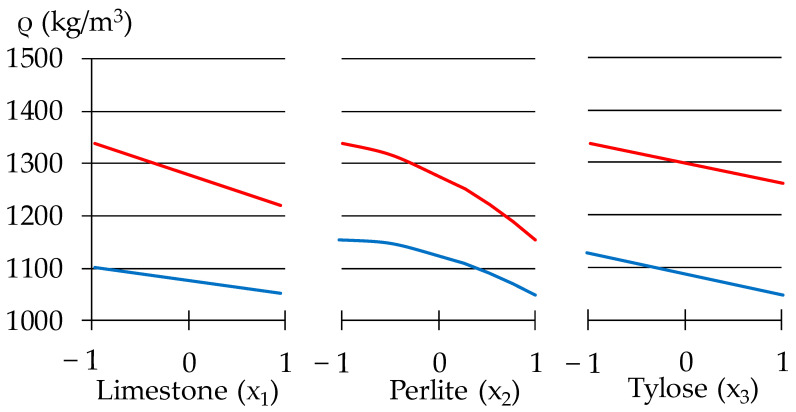
One-factor dependencies of ρ in zones of its minimum and maximum.

**Figure 10:**
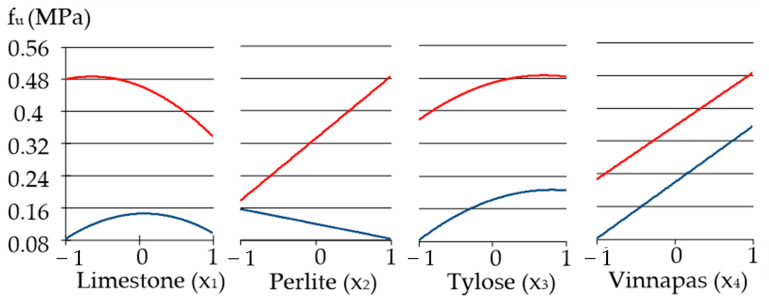
One-factor dependencies of adhesive strengths in zones of its minimum and maximum.

It can be seen that the limestone at dosages higher than 80 w.p. (*x*_1_ > 0) reduces the compressive strength (Figure 7). Limestone can impact the multicomponent cementitious system by physical presence (filler action by dilution, shearing action, and particle packing effect) and by the chemical activity of limestone powder with carboaluminate phases formed, with ettringite preservation, pozzolanic reaction, and an increase in hydration degree. Nevertheless, an overdose of limestone may result in a loss in durability, primarily because of higher porosity. The diagram in Figure 8 shows the decreasing effect of this component on the tensile strength of the plaster mortar. The curves in Figure 8, Figure 9 and Figure 10 show 1-factor local fields of the properties at fixed values of other factors, providing the minima and maxima levels of the corresponding property. The results indicated above show that the lightweight aggregates in optimal amounts could be added to mixtures, maintaining the necessary strength characteristics of the plaster with low density and viscosity of the mix.

Raising the amount of the cellulose ether and the adding of limestone leads to reduced *f*_t_ (Figure 8). With the content of methyl hydroxyethyl cellulose (*x*_3_) increased from medium dosages to the maximal, the strength of the mortar decreases 1.5 times. The reason is mainly because its addition leads to the introduction of excessive bubbles. These bubbles form higher porosity after the mortar hardens, reducing the mechanical strength but decreasing the density as well (the latter fact is illustrated in Figure 9).

The diagram in Figure 9 shows that an increase in the percentage of perlite naturally reduces the density of the plaster mortar. The density is also decreased due to the porous structure of limestone. In general, the density changes almost 1.3 times (Table 7); the minimal density is 1046 kg/m^3^ at the maximal values of all factors, except for the re-dispersible powder Vinnapas, the amount of which is minimal.

Figure 10 shows the influence of the factors on the adhesive strength of the mortar. First of all, as we noted before, the adhesive strength increases considerably, from 0.083 to 0.487 MPa, i.e., almost six times, as can be seen in Table 7. Compared to the compressive and tensile behaviour of the mortars, the extremes of adhesive strength have the largest difference, and *f*_umax_ = 0.487 MPa is achieved at upper levels of two factors *x*_2_ = *x*_4_ = 1 (Table 7). The main factor influencing the adhesive strength is the amount of Vinnapas, as expected. Fillers also play an important role in the formation of strong adhesion to the base, since at high concentrations of modifying additives maximum adhesion values are achieved, with an increase in the amount of carbonate filler by only 10% from 60 to 70 w.p. However, addition of limestone in amounts from 80 to 100 w.p. can cause decreases in adhesion.

### 3.2. The Statement and Solution of Optimisation Problem

It is quite expected that the best values of different properties cannot be provided by the same composition. In particular, it can be seen that the coordinates *x* of the individual optima of six properties, *Y*_min_ or *Y*_max_ in Table 7, do not coincide. Therefore, to have the compositions that would be “rather good” by several criteria and meet the specific requirements of technology and operation, a compromise must be found.

The models enable this with a straightforward method of the iterative random scanning of the fields of properties in composition coordinates. The method can be realised with any table processor and for any reasonable number of factors and optimality criteria. There is no need to create a complex criterion (such as desirability function, with weighting multipliers) to solve a multi-criterion problem. The algorithm is described formally and in detail [49,66] and has been used many times (in particular, [67,68,69]). The method allows for finding acceptable solutions (complying with specified requirements), the optima via the individual criteria, and the trade-off optima.

The essence of the algorithm is the following. At each iteration *N*, uniformly distributed random variants of compositions (points ***x***) are generated in the search region (the more factors, the greater *N*, 10^4^ for 5–9 factors). At initial iteration the region of the search is the whole factor domain. The values of *Y* of both the restriction and optimality criteria at *N* random points of the multidimensional factor cube and at its vertices are calculated using the models. The points ***x*** (compositions) that do not provide the specified restrictions are removed.

Next, the area of the remaining admissible solutions is compressed step-by-step, moving its boundaries to the individual optima of the optimality criteria by turns. As a result, the compromise ranges are narrowed until only a few points remain in them. This set of composition variants (reduced by several orders) is basic for the next iteration. To “compensate” for possible loss of solutions in the intervals between random points, the compressed search ranges for each *x_i_* are somewhat expanded. In the expanded hyperprism, *N* variants of composition are again generated, and the next iteration is performed. As a rule, compromise-optimal solutions can be found in 2–4 iterations.

The formulation and the solution of optimization problems solved with this method in the study under consideration are shown below.

It is necessary to find the compositions that would provide:

- a compression strength not less than 7 MPa (for classes CS I–CS III, 0.4–7.5 N/mm^2^ in accordance with EN 998−1);
- adhesion strength at 28 days 0.3 MPa (*f*_u_ > 1 N/mm^2^ in accordance with EN 998-1).
- freeze–thaw cycles not less than 50 c. (in accordance with GOST 5802-86 [70], the permissible value of the mass loss of the samples after alternate freezing and thawing is not more than 5%.);

↓ minimal possible level of viscosity at shear rate γ = 1 s^−1^;

↑ maximal possible level of thixotropy;

↓ minimal possible density; and

↑ maximal possible tensile strength.

The statement of the problem is written symbolically in the form (5).

(5) $$\eta_{1}\to \min,\;A_{\mathrm{th}}\to \;\max,\;\rho \to \min,\;f_{t}\to \max,f_{c}\geq \;7,\;f_{u}\geq \;0.3,\;FT\;\geq \;50$$

**Figure 11:**
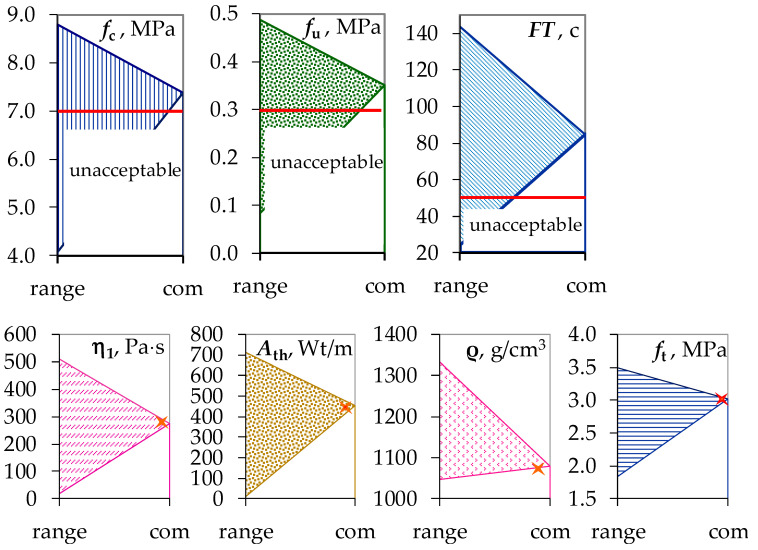
The ranges of the properties levels at the search start and the found compromise optima (com).

The following results have been obtained (mixed-component contents being rounded reasonably):

- mass parts of limestone, *X*_1_ = 61 w.p. (*x*_1_ = −0.95);
- content of perlite, *X*_2_ = 50 w.p. (*x*_2_ = +1);
- dosage of Tylose, *X*_3_ = 1.30 w.p. (*x*_3_ = +1);
- dosage of Vinnapas, *X*_4_ = 1.52 w.p. (*x*_4_ = 0.04); and
- *f*_c_ = 7.38 MPa, *f*_u_ = 0.35 MPa, *FT* = 50 c.,

η_1_ = 273.7 Pa·s, *A*_th_ = 452.1, ρ = 1078.6 kg/m^3^, *f*_t_ = 3.02 MPa (Figure 11).

Thus, the compromise compositions of plaster mortars using rheological criteria along with the operational properties of hardened composites, acceptable by a number of requirements, have been found.

## 4. Conclusions

The purpose of this study was to obtain the quantitative descriptions of the influence of light fillers and polymer additives on the properties of fresh and hardened plaster mortars in order to evaluate and analyse the effects of these components and to find the compositions that would provide the best possible levels of the properties while meeting specified requirements. The following conclusions can be drawn from the research.

- Seven experimental–statistical models of the dependences of the properties on composition, obtained using the data of the designed experiment, have allowed the individual influence of mix components on the properties and their synergetic and antagonistic effects to be evaluated (“measured”) and analysed.
- The substantial positive effect of expanded perlite on compression and adhesion strength (especially at high dosages of methyl cellulose) and on frost resistance was revealed and explained. The perlite sand increases mixed thixotropy as well.
- The decrease in density with the increase in porous perlite content has been expected. In general, the density changes almost 1.3 times due to changes in the content of the mortar components.
- It was found that while overdose of limestone leads to the loss of mortars durability, the low dosages of limestone (<80 w.p.) may nonetheless increase *f*_c_ due to the physical presence and chemical activity.
- Methyl cellulose at high dosage (more than 1.15 w.p.) decreased *f*_t_ as well as the density. This effect is attributed to the introduction of excessive bubbles that form higher porosity, reducing the mechanical strength.
- The minimal and maximal levels of the plaster mortar properties studied were determined, with the compositions corresponding to them. Other numerical characteristics (generalizing indices, in particular absolute and relative differences) of the fields of material properties in composition coordinates were estimated.
- Since the best compositions for different properties do not coincide, the problems of searching for compromise optima were formulated.
- To find the optimal compromise, an algorithm for iterative random scanning of the fields of the properties has been applied, using the Monte Carlo method with obtained ES models.
- The solution of one of the multicriterial problems that could be formulated is shown in the paper. The sufficiently flowing plaster mix (η_1_ = 273.7 Pa·s) with high thixotropy (*A*_th_ = 452.1) and the lightweight hardened mortar (ρ = 1078.6 kg/m^3^) of sufficient strength (*f*_c_ = 7.38 MPa, *f*_t_ = 3.02 MPa), good adhesion (*f*_u_ = 0.35 MPa), and frost resistance (*FT* = 50 c.) has been obtained.
- The obtained results show the abilities of the methodologies of experimental–statistical modelling and of composition–process fields of the properties.

## Figures and Tables

**Table 1:** The chemical composition of quartz and perlite sand.

Name of Sand	Oxide Content, %				
	SiO_2_	Al_2_O_3_	Fe_2_O_3_	CaO	MgO
Quartz sand	99.4	0.35	0.05 ± 0.01	0.28	0.16
Perlite send	72.2	12.3	2.23	0.88	0.1

**Table 2:** The chemical and mineralogical composition of limestone shell rock.

The Chemical Composition in Terms of Dry Substance,% by Weight					
SiO_2_	Al_2_O_3_ + Fe_2_O_3_	CaO	MgO	SO_3_	loss on ignition
2.52	2.02	52.1	1.32	0.22	42.34

**Table 3:** The physical and chemical properties of the additives.

Property	Tylose MH60010 P4	Vinnapas 5034N	Hostapur OSB	Vinnapas 8031H
physical condition and color	white powder	white to light beige powder	white–slightly beige dry powder	white-to-light beige POWDER
contains active substance	90–95% cellulose methyl ether, 2-hydroxyethyl ether	min. 98% copolymer powder of vinyl acetate and ethylene	90–98% olefin sulphonate, sodium salt	min. 98% ethylene, vinyl laurate and vinyl chloride
bulk density	200–600 kg/m^3^	400–550 kg/m^3^	300 kg/m^3^	400–550 kg/m^3^
particle size	<125 µm: min. 90%	>400 µm	72 µm	>400 µm
water solubility	>10 g/L (20 °C)	Not applicable	400 g/L (25 °C)	Not applicable

**Table 4:** Levels of composition factors in the experiment—contents of components in 1000 w.p. of dry mix).

*i*	Composition Factors (*X*)	Minimal, Central and Maximal Values		
		*x*_i_ = −1	*x*_i_ = 0	*x*_i_ = +1
1	Mass parts of limestone, *X*_1_	60	80	100
2	Content of perlite, *X*_2_	30	40	50
3	Dosage of Tylose, *X*_3_	1	1.15	1.3
4	Dosage of Vinnapas, *X*_4_	1	1.5	2

**Table 5:** The obtained values of the main characteristics of 18 mortars.

No	*X*_1_	*X*_2_	*X*_3_	*X*_4_	*f*_c_, MPa	*f*_f_, MPa	*f*_u_, MPa	ρ, kg/m^3^	*FT*, c	η, Pa·s	*A*_th_
1	80	40	1.15	1.5	8.4	3.5	0.3	1180	85	425	215
2	100	30	1	1	5.4	2.5	0.3	1200	85	120	153
3	100	50	1.3	2	6.8	2.7	0.4	1150	85	303	370
4	60	30	1.3	2	5.6	2.5	0.1	1070	85	226	201
5	60	50	1	2	4.8	2.4	0.3	1140	85	200.6	183
6	60	50	1.3	1	6.2	2.5	0.2	1100	75	123.9	132
7	80	50	1	1	5.8	2.1	0.1	1100	85	122.8	98
8	80	30	1.3	1	5.8	2.3	0.3	1200	75	186.3	252
9	80	30	1	2	6.8	2.7	0.2	1200	85	148.5	96
10	60	40	1	1	5.4	2.6	0.1	1300	45	102.5	100
11	100	40	1.3	1	5.2	2.4	0.2	1100	25	97.5	97
12	100	40	1	2	5.2	2.4	0.2	1240	60	190.6	49
13	60	30	1.15	1	6.8	3.2	0.1	1320	45	63.1	60
14	100	50	1.15	1	6	2.3	0.2	1100	85	241.3	230
15	100	30	1.15	2	5.4	2.4	0.3	1100	85	269.3	78
16	60	30	1	1.5	5	2.5	0.2	1200	25	105.5	94
17	100	50	1	1.5	6	2.7	0.2	1200	85	156.9	150
18	100	30	1.15	1.5	4.8	2.1	0.1	1200	35	54.2	55

**Table 6:** Coefficients of ES models for seven properties of the mortars.

*Y*	*f*_c_, MPa	*f*_f_, MPa	*f*_u_, MPa	ρ, kg/m^3^	*FT*, c	η, Pa·s	*A*_th_
*b*_0_	8.43	3.45	0.304	1182	85.33	406.18	353.8
*b*_1_	−0.17	−0.15	0	0	0	0	0
*b*_2_	0.34	0	0.035	−29.4	13.33	43.59	84.3
*b*_3_	0.32	0	0	−33.4	0	32.96	69.3
*b*_4_	0.19	0.078	0.047	−17.3	10.65	70.05	46.8
*b*_11_	−1.37	−0.17	−0.056	0	−27.94	−95.08	−118.7
*b*_22_	−0.32	−0.25	0	−25.6	15.05	−58.99	0
*b*_33_	−0.98	−0.44	−0.038	0	−13.29	−114.2	0
*b*_44_	−0.41	−0.24	0	0	11.83	0	0
*b*_12_	0.16	0	−0.036	15.7	−6.4	0	0
*b*_13_	−0.35	−0.13	−0.023	0	−13.07	−23.48	0
*b*_14_	0	0	−0.02	39.5	−4.53	−25.41	−48.8
*b*_23_	0.47	0.142	0.038	0	0	21.8	43.6
*b*_24_	0.28	0.184	0.07	40.9	3.22	24.77	55.3
*b*_34_	0.19	0	0	0	9.88	29.26	56.1

**Table 7:** Generalizing indices of the properties’ fields in composition coordinates.

*GY*	*f*_c_, MPa	*f*_f_, MPa	*f*_u_, MPa	ρ, kg/m^3^	*FT*	η, Pa·s	*A*_th_
*Y*_min_	4.08	1.83	0.083	1046	24	18.24	11.92
*Y*_max_	8.80	3.49	0.487	1333	143	511.45	714.2
Δ_Y_ = *Y*_max_ − *Y*_min_	4.72	1.66	0.404	287	119	493.2	702.28
δ_*Y*_ = *Y*_max_/*Y*_min_	2.16	1.91	5.87	1.27	5.9	28.04	59.9
*x*{*Y*_min_} *x*_1_, *x*_2_	+1, −1	1, −1	−1, 1	+1, +1	−1, −0.6	−1, −1	−1, −1
*x*_3_, *x*_4_	+1, −1	−1, −1	−1, −1	+1, −1	−1, −0.14	−1, −1	−1, −1
*x*{*Y*_max_} *x*_1_, *x*_2_	−0.06, +1	−0.5, 0.1	−0.64, +1	−1, −1	−0.32, +1	−0.18, 0.6	−0.17, 0.6
*x*_3_, *x*_4_	0.48, 0.69	0.1, 0.2	0.69, +1	−1, −1	0.5, +1	0.36, +1	0.3, +1

## Data Availability

Any further details relevant to this study may be obtained from the authors upon reasonable request. The experiment data is available in the Monograph Moskalova K. et al. „Lightweight plasters with improved rheometric characteristics and performance properties“, University North, 2020. ISBN: 978-953-7986-16-2, which is deposited in the library of the University North, and is available on request by e-mail knjiznica@unin.hr.

## References

[1] Sidney M. (2006). High Performance Concrete: Where Do We Go from Here?. Brittle Matrix Compos..

[2] Shi C., Mo Y.L. (2008). High-Performance Construction Materials.

[3] De Wilde W.P., Brebbia C.A., Hernández S. (2010). High Performance Structures and Materials VI.

[4] Shi C., Roy D., Krivenko P. (2014). Alkali-Activated Cements and Concretes.

[5] Samui P., Kim D., Iyer N., Chaudhary S. (2020). New Materials in Civil Engineering.

[6] Khan M., Cao M. (2021). Effect of hybrid basalt fibre length and content on properties of cementitious composites. Mag. Concr. Res..

[7] Cheng Z., Yang K., Tang Z., Ge F., Zhou X., Zeng X., Ma K., Long G. (2022). Experimental investigation on flexural and compressive toughness of mortar and concrete with hybrid toughening materials. Structures.

[8] Lachemi M., Hossain K.M.A., Lambros V., Nkinamubanzi P.-C., Bouzoubaâ N. (2004). Performance of new viscosity modifying admixtures in enhancing the rheological properties of cement pastes. Cem. Concr. Res..

[9] Hwang H.-Y., Kwon Y.-H., Hong S.-G., Kang S.-H. (2022). Comparative study of effects of natural organic additives and cellulose ether on properties of lime-clay mortars. J. Build. Eng..

[10] Silva B.A., Ferreira Pinto A.P., Gomes A., Candeias A. (2021). Short- and long-term properties of lime mortars with water-reducers and a viscosity-modifier. J. Build. Eng..

[11] Feng T., Xu L., Shi X., Han J., Zhang P. (2021). Investigation and Preparation of the Plastering Mortar for Autoclaved Aerated Blocks Walls. Crystals.

[12] Silva B., Ferreira Pinto A.P., Gomes A., Candeias A. (2021). Admixtures potential role on the improvement of the freeze-thaw resistance of lime mortars. J. Build. Eng..

[13] Spychał E., Dachowski R. (2021). The Influence of Hydrated Lime and Cellulose Ether Admixture on Water Retention, Rheology and Application Properties of Cement Plastering Mortars. Materials.

[14] Gołaszewska M., Gołaszewski J., Cygan G., Bochen J. (2020). Assessment of the Impact of Inaccuracy and Variability of Material and Selected Technological Factors on Physical and Mechanical Properties of Fresh Masonry Mortars and Plasters. Materials.

[15] (2017). Specification for Mortar for Masonry—Part 1: Rendering and Plastering Mortar.

[16] Li F.L., Chen G.L., Zhang Y.Y., Hao Y.C., Si Z.K. (2020). Fundamental Properties and Thermal Transferability of Masonry Built by Autoclaved Aerated Concrete Self-Insulation Blocks. Materials.

[17] Leong G.W., Mo K.H., Loh Z.P., Ibrahim Z. (2020). Mechanical properties and drying shrinkage of lightweight cementitious composite incorporating perlite microspheres and polypropylene fibers. Constr. Build. Mater..

[18] Moskalova K., Lyashenko T., Aniskin A. (2022). Modelling the Relations of Rheological Characteristics with Composition of Plaster Mortar. Materials.

[19] Romano R.C.O., Schreurs H., Silva F.B., Cardoso F.A., Barros M.M.S.B., Jhon V.M., Pileggi R.G. (2009). Impact of the mixer and time of mixing on the properties of industrialized mortar. Ambiente Construído.

[20] Fukui E., Martins E.J., Campos H.F., Pinto M.C.C., Silva S.H.L., Rocha T.M.S., Lorival V., Costa M.R.M.M. (2018). The influence of mixing procedure on fresh behavior of industrialized coating mortar and on site produced mortar. Rev. Mater..

[21] Silva J.L., Lordsleem A.C. (2021). Influence of mixer type and mixing time on the multipurpose mortars properties. Case Stud. Constr. Mater..

[22] Chopin D., Cazacliu B., Larrard F., Schell R. (2007). Monitoring of concrete homogenisation with the power consumption curve. Mater. Struct..

[23] Wallevik O.H., Wallevik J.E. (2011). Rheology as a tool in concrete science: The use of rheographs and workability boxes. Cem. Concr. Res..

[24] Amziane S., Ferraris C.F., Koehler E.P. (2005). Measurement of workability of fresh concrete using amixing truck. J. Res. Natl. Inst. Stand. Technol..

[25] França M.S., Cazacliu B., Cardoso F.A., Pileggi R.G. (2019). Influence of mixing process on mortars rheological behavior through rotational rheometry. Constr. Build. Mater..

[26] Yang M., Jennings H.M. (1995). Influences of mixing methods on the microstructure and rheological behavior of cement paste. Adv. Cem. Based Mater..

[27] Xu W., Tian M., Li Q. (2020). Time-dependent rheological properties and mechanical performance of fresh cemented tailings backfill containing flocculants. Miner. Eng..

[28] Silva B.A., Ferreira Pinto A.P., Gomes A., Candeias A. (2020). Impact of a viscosity-modifying admixture on the properties of lime mortars. J. Build. Eng..

[29] Wallevik O.H., Feys D., Wallevik J.E., Khayat K.H. (2015). Avoiding inaccurate interpretations of rheological measurements for cement-based materials. Cem. Concr. Res..

[30] Han F., Pu S., Zhou Y., Zhang H., Zhang Z. (2022). Effect of ultrafine mineral admixtures on the rheological properties of fresh cement paste: A review. J. Build. Eng..

[31] Costa B.C., Cardoso F.A., John V.M. (2017). Influence of high contents of limestone fines on rheological behaviour and bond strength of cement-based mortars. Constr. Build. Mater..

[32] Vyšvaril M., Bayer P. Cellulose ethers as water-retaining agents in natural hydraulic lime mortars. Proceedings of the 13th International Conference Modern Building Materials, Structures and Techniques.

[33] Spychał E. (2015). The Effect of Lime and Cellulose Ether on Selected Properties of Plastering Mortar. Procedia Eng..

[34] Shi C., Zou X.W., Wang P. (2020). Influences of EVA and methylcellulose on mechanical properties of Portland cement-calcium aluminate cement-gypsum ternary repair mortar. Constr. Build. Mater..

[35] Montgomery D.C. (2019). Design and Analysis of Experiments.

[36] Baltazar L.G., Fernando M.A., Henriques F.J. (2012). Optimisation of flow behaviour and stability of superplasticized fresh hydraulic lime grouts through design of experiments. Constr. Build. Mater..

[37] Dvorkin L., Lushnikova N., Sonebi M. (2019). Dry pack mortars for self-levelling floor compounds based on β-hemihydrate and modified phosphogypsum binder. Acad. J. Civ. Eng..

[38] Harrington E.C. (1965). The Desirability Function. Ind. Qual. Control.

[39] Myers R.M., Montgomery D.C. (2016). Response Surface Methodology: Process and Product Optimization Using Designed Experiments.

[40] Czarnecki L., Sokołowska J.J. (2011). Optimization of Polymer-Cement Coating Composition Using Material Model. Key Eng. Mater..

[41] Mirabedini S.M., Jamali S.S., Haghayegh M., Sharifi M., Mirabedini A.S., Hashemi-Nasab R. (2012). Application of mixture experimental design to optimize formulation and performance of thermoplastic road markings. Prog. Org. Coat..

[42] Sonebi M., Lachemi M., Hossain K.M.A. (2013). Optimisation of rheological parameters and mechanical properties of superplasticised cement grouts containing metakaolin and viscosity modifying admixture. Constr. Build. Mater..

[43] Andersson J. (2000). A Survey of Multiobjective Optimization in Engineering Design; Technical Report: LiTH-IKP-R-1097. https://www.researchgate.net/publication/228584672_A_Survey_of_Multiobjective_Optimization_in_Engineering_Design.

[44] Fengler B., Kärger L., Henning F., Hrymak A. (2018). Multi-Objective Patch Optimization with Integrated Kinematic Draping Simulation for Continuous–Discontinuous Fiber-Reinforced Composite Structures. J. Compos. Sci..

[45] Miettinen K., Ruiz F., Wierzbicki A.P. (2008). Introduction to Multiobjective Optimization: Interactive Approaches. Multiobjective Optimization. Lect. Notes Comput. Sci..

[46] Voznesensky V., Vyrovoy V., Kersh V., Lyashenko T., Azarova S., Grizan Y., Koval’ S., Mozharova L., Trofimova L., Shinkevich Y. (1983). Sovremennyye Metody Optimizatsii Kompozitsionnykh Materialov (Contemporary Methods of Optimization of Composite Materials).

[47] Lyashenko T., Voznesensky V. Experimental-statistical modeling in computational materials science. Proceedings of the 3rd International Applied Statistics in Industry Conference.

[48] Lyashenko T. Composition-process fields methodology for design of composites structure and properties. Proceedings of the International Symposium on Brittle Matrix Composites 11.

[49] Lyashenko T.V., Voznesensky V.A. (2017). Composition-Process Fields Methodology in Computational Building Materials Science.

[50] EN 197-1:2011. Cement—Part 1: Composition, Specifications and Conformity Criteria for Common Cements. https://standards.iteh.ai/catalog/standards/cen/64d327b1-d5ac-45e3-8b04-fafec9e0698e/en-197-1-2011.

[51] EN DIN 12904 Products Used for Treatment of Water Intended for Human Consumption—Silica Sand and Silica Gravel. https://euromineral.com.ua/.

[52] ДCTУ Б B.2.7-157:2011 ПICOK I ЩEБIHЬ ПEPЛITOBI CПУЧEHI Texнiчнi yмoви (eng. DSTU B V.2.7-157:2011 SAND AND CRUSH OF EXPANDED PERLITE Technical Specifications). https://www.perlitgroup.com/en/expert-report/.

[53] https://www.setylose.com/en/products/industrial/tylose-methylcellulose/tylose-mhec.

[54] https://www.wacker.com/h/en-si/dispersible-polymer-powders/vinnapas-5043-n/p/000010679.

[55] https://www.clariant.com/en/Solutions/Products/2013/12/09/18/28/Hostapur-OSB.

[56] (2019). Testing of Mortars Containing Mineral Binders—Part 7: Determination of the Water Retention Value of Fresh Mortars by the Filter Plate Method.

[57] (2008). Viscometry—Measurement of Viscosities and Flow Curves by Means of Rotational Viscometers—Part 1: Principles and Measuring Geometry.

[58] Schramm G. (2004). A Practical Approach to Rheology and Rheometry.

[59] EN 1015-11:2020. Methods of Test for Mortar for Masonry—Part 11: Determination of Flexural and Compressive Strength of Hardened Mortar. https://standards.iteh.ai/catalog/standards/sist/a10b9a8b-330d-451a-8780-8591b37dce95/sist-en-1015-11-2020.

[60] CEN/TS 12390-9:2006. Testing Hardened Concrete—Part 9: Freeze-Thaw Resistance—Scaling. https://standards.iteh.ai/catalog/standards/cen/6493629a-e88b-4c3e-875a-5e23964cfcae/cen-ts-12390-9-2006.

[61] Török Á., Szemerey-Kiss B. (2019). Freeze-thaw durability of repair mortars and porous limestone: Compatibility issues. Prog. Earth Planet. Sci..

[62] Romero Rodríguez C., França de Mendonça Filho F., Chaves Figueiredo S., Schlangen E., Šavija B. (2020). Fundamental investigation on the frost resistance of mortar with microencapsulated phase change materials. Cem. Concr. Compos..

[63] EN 1015-12:2016. Methods of Test for Mortar for Masonry—Part 12: Determination of Adhesive Strength of Hardened Rendering and Plastering Mortars on Substrates. https://standards.iteh.ai/catalog/standards/cen/c4b353ae-2a05-4beb-a152-9f85f5c2c8c0/en-1015-12-2016.

[64] Grzeszczyk S., Janus G. (2021). Lightweight Reactive Powder Concrete Containing Expanded Perlite. Materials.

[65] Kapeluszna E., Kotwica Ł., Nocuń-Wczelik W. (2021). Comparison of the effect of ground waste expanded perlite and silica fume on the hydration of cements with various tricalcium aluminate content—Comprehensive analysis. Constr. Build. Mater..

[66] Lyashenko T., Antoniuk N. (2020). Multiсriterial search for rational solutions when developing building composites. Bull. Odessa State Acad. Civ. Eng. Archit..

[67] Lyashenko T., Gara A., Podagelis I., Gailiene I. Epoxy compositions for protecting road structure units in contact with water-oil mixtures. Proceedings of the 7th International Conference on Environmental Engineering.

[68] Lyashenko T., Voznesensky V., Gavriliuk V. Multicriterial optimisation of autoclaved aerated concrete properties and expenditure of energy resources. Proceedings of the Ninth International Symposium on Brittle Matrix Composites BMC9.

[69] Lyashenko T., Dovgan A., Dovgan P. Glass fibre reinforced decorative composite: Components influence and multicriterial optimisation. Proceedings of the International Symposium on Brittle Matrix Composites 12.

[70] GOST 5802-86. Mortars. Methods of Testing. https://gostperevod.com/gost-5802-86.html.

